# Late-Onset Scleral Flap Injury After Trabeculectomy Successfully Repaired Using a Preserved Scleral Patch: A Report of Two Cases

**DOI:** 10.7759/cureus.97267

**Published:** 2025-11-19

**Authors:** Shuzo Okuno, Koji Namiguchi, Tomoko Naito, Shiro Mizoue, Atsushi Shiraishi

**Affiliations:** 1 Department of Ophthalmology, Ehime University Graduate School of Medicine, Toon, JPN; 2 Department of Ophthalmology, Grace Eye Clinic, Okayama, JPN

**Keywords:** filtration surgery, preserved sclera, primary open angle glaucoma, scleral damage, trabeculectomy

## Abstract

Scleral flap repair may be required during or after filtration surgery or tube shunt surgery. This report discusses two cases wherein preserved scleral patching was successfully used to treat scleral flap damage that occurred after combined use with mitomycin C (MMC) trabeculectomy.

The first case was a 59-year-old female who sustained right-eye trauma, resulting in conjunctival damage, a scleral flap defect, and prolapse of the choroid under the conjunctiva. She had a history of trabeculectomy for primary open-angle glaucoma (POAG) in both eyes five years ago. This patient was treated by attaching a preserved scleral patch to the site of the scleral flap defect, resulting in good intraocular pressure (IOP) and no complications. The second case was a 76-year-old male who consulted for right vision loss without any identifiable cause and was found to have avascular bleb damage, aqueous humor leakage, scleral flap melting, and choroidal detachment. He had a previous history of trabeculectomy for POAG in both eyes three years ago, as well as a right-bleb infection treated with intravitreal antibiotic injection and conjunctival resection two years ago. This patient was treated with a preserved scleral patch to the damaged area, plus conjunctival advancement. The aqueous humor leakage and choroidal detachment improved after surgery. However, the IOP remained elevated (approximately 20 mmHg), and this was successfully treated by inserting an Ahmed Glaucoma valve™ (FP7, New World Medical, Inc., Rancho Cucamonga, CA, USA). These cases demonstrate that the application of a preserved scleral patch may be viable for late-onset flap injuries after glaucoma surgery.

## Introduction

Glaucoma is the leading cause of visual impairment in Japan [1], and its management includes medications, laser therapy, and surgical interventions, such as filtration surgery. Since Sugar’s initial report in 1961 [2], trabeculectomy has remained the most commonly performed glaucoma procedure. In this surgery, aqueous humor is diverted through a surgically created pathway involving the iris and trabecular meshwork, passing beneath a scleral flap into a filtering bleb under the conjunctiva. To reduce postoperative scarring and maintain bleb function, mitomycin C (MMC) is often applied intraoperatively.

Despite its effectiveness, filtration surgery carries risks such as bleb infection, endophthalmitis [3], overfiltration, and traumatic damage. Scleral flaps and tunnels play a critical role in controlling aqueous outflow and preventing tube exposure in both trabeculectomy and tube shunt surgery. However, scleral flap damage may occur intraoperatively or long after surgery, due to infection, trauma, or excessive filtration. Previous reports have described cases of flap thinning, melting, or rupture following bleb infection, trauma, or even the Valsalva maneuver [4].

Corneal grafts and autologous scleral grafts are established options for repairing such defects, but preserved scleral patches have been less frequently reported despite their advantages: they are readily available, provide sufficient strength, and can cover large defects. Late-onset scleral flap melting is rare but clinically significant, as it can lead to severe complications, including hypotony, infection, or even loss of the eye if untreated.

## Case presentation

Case 1

**Figure 1:**
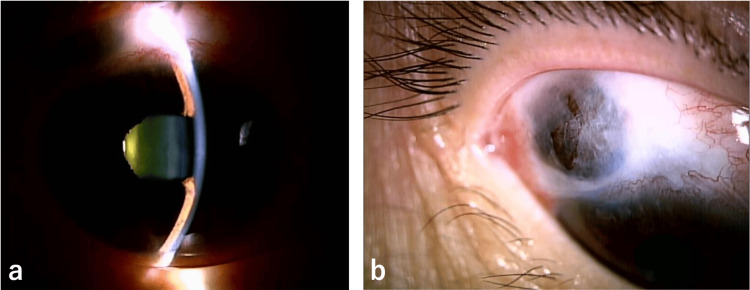
Slit-lamp photographs at the initial consultation, revealing shallowing of the anterior chamber depth (a) and prolapse of the choroid into the subconjunctival space (b).

A 59-year-old woman presented with right eye pain and vision loss after being struck by a support post while working in the fields. Five years prior, she underwent trabeculectomy using 0.04% MMC for a three-minute surgery on both eyes for bilateral primary open-angle glaucoma (POAG). Upon examination, the right eye had a best-corrected logMAR visual acuity of 0.7 and an intraocular pressure (IOP) of 3 mmHg. There were defects in the scleral flap, with prolapse of choroid into the subconjunctival space, resulting in aqueous humor leakage and loss of the anterior chamber (Figure 1). The crystalline lens remained intact, and although choroidal detachment was observed in the inferior fundus, no retinal detachment was identified.

**Figure 2:**
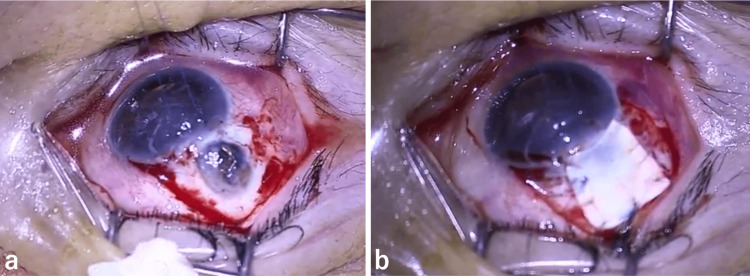
Intraoperative photographs showing prolapse of the choroid (a), covered by the scleral patch (b).

Two days of conservative treatment with rest, eye drops (0.5% moxifloxacin, 0.5% ofloxacin eye ointment, and 1% atropine), and oral acetazolamide failed to improve the aqueous humor leakage and anterior chamber depth; thus, repair surgery was performed. During surgery, the filtering bleb was incised at the base of the fornix to expose the sclera, and the preserved sclera was trimmed and sutured with 10-0 nylon thread to adequately cover the choroidal herniation (Figure 2).

**Figure 3:**
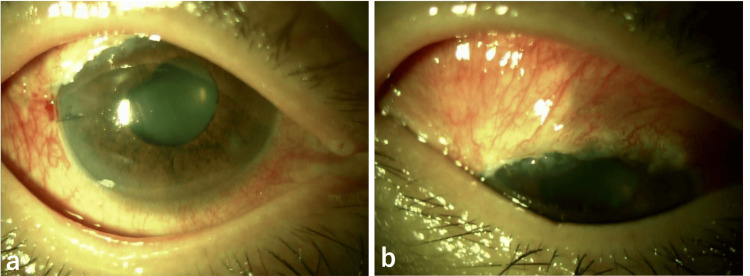
Postoperative photographs showing a normal anterior chamber depth (a) and no leakage (b).

To complete the procedure, the conjunctiva was rotated forward and sutured with 10-0 nylon thread. On postoperative day 1, 0.1% betamethasone sodium phosphate eye drops were administered four times daily, and 0.5% moxifloxacin eye drops six times daily. The IOP was 26 mmHg. As the IOP remained elevated on postoperative day 3, a 0.1% dorzolamide/0.05% timolol fixed-combination eye drop was added. Following this adjustment, the IOP decreased to approximately 15 mmHg. The anterior chamber depth remained consistently normal throughout the postoperative period, and no aqueous humor leakage was observed (Figure 3).

At two-year follow-up, the right eye maintained an IOP of 12 mmHg and a corrected logMAR visual acuity of 1.2, with glaucoma eye drops (0.1% dorzolamide/0.05% timolol fixed-combination eye drop).

Case 2

**Figure 4:**
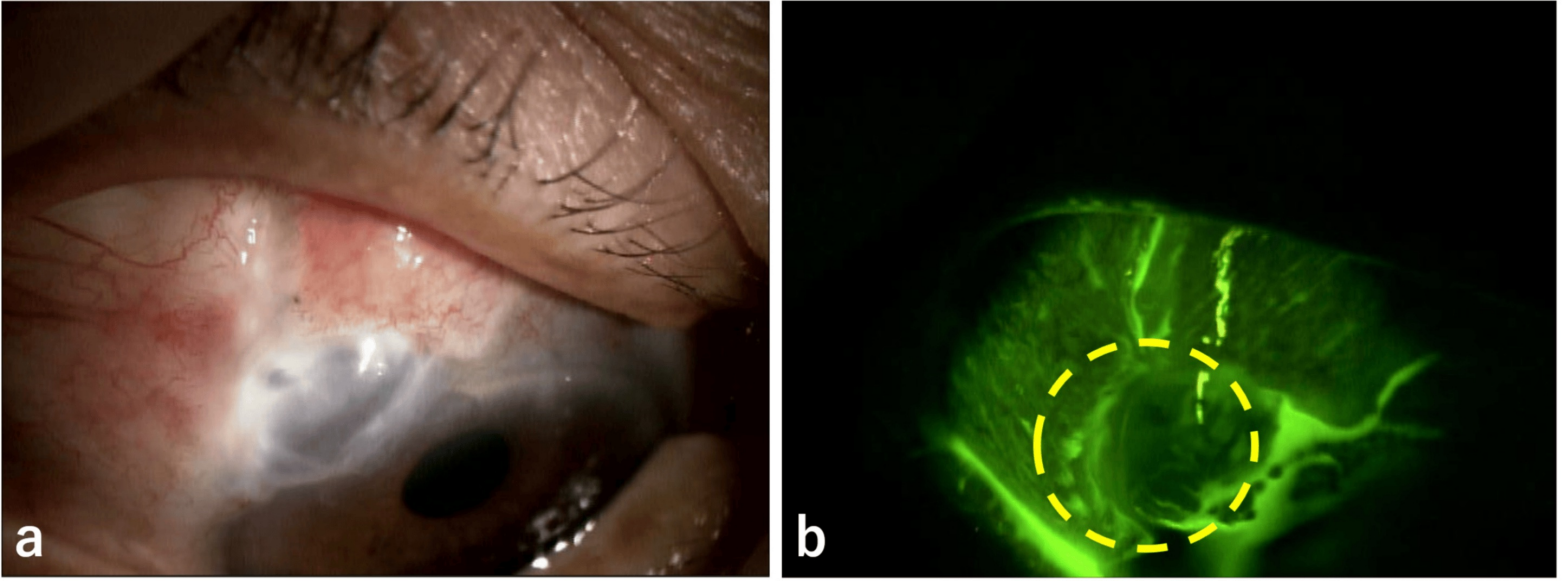
Slit-lamp photographs from the first consultation showing leakage (b) from the avascular bleb (a).

A 76-year-old male sought consultation at a nearby hospital for decreased visual acuity in the right eye. He was referred to our hospital because the right eye was found to have poor corrected logMAR visual acuity (0.4), an IOP of 4 mmHg, aqueous humor leakage from an avascular filtering bleb, scleral flap melting, and choroidal detachment (Figure 4). The anterior chamber depth was maintained throughout, the intraocular lens remained stable, and choroidal detachment was observed in the fundus.

**Figure 5:**
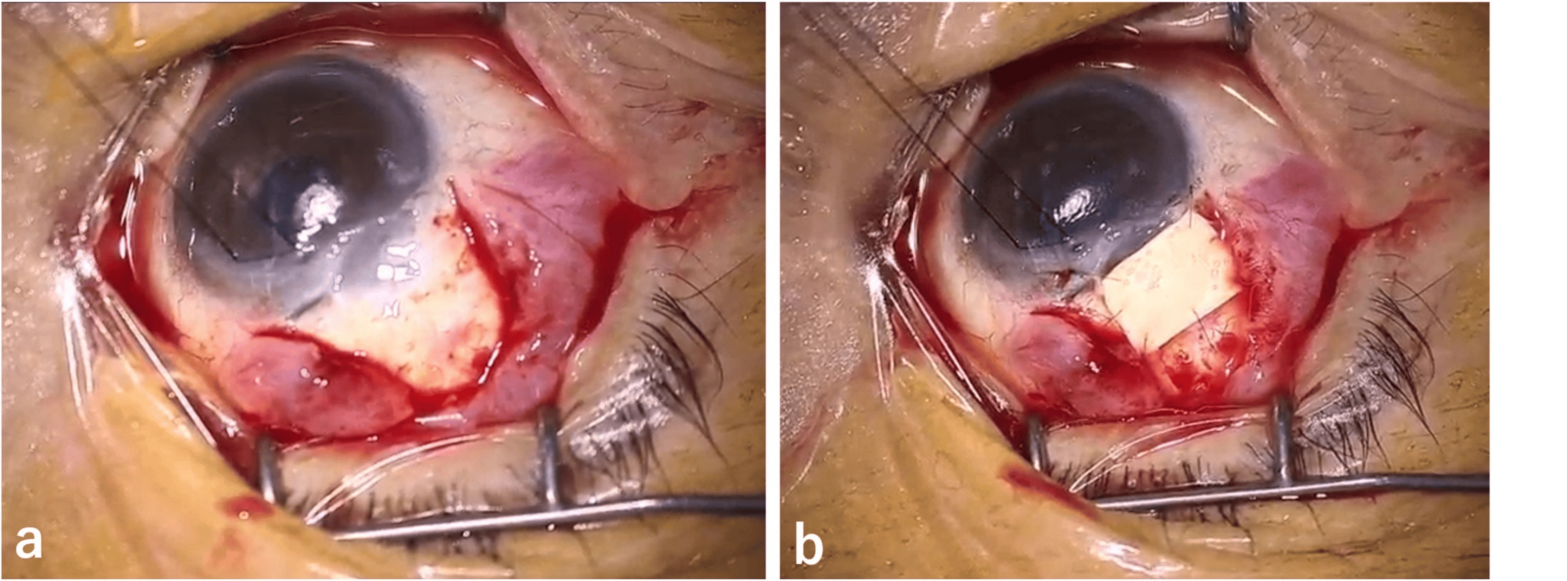
Intraoperatively, a preserved scleral patch (b) was applied to the area of the dissolved scleral flap (a).

Three years ago, he underwent bilateral supratemporal trabeculectomy using 0.04% MMC for a three-minute surgery for POAG in both eyes. Two years ago, he had a filtering bleb infection of the right eye, which was managed with anterior chamber irrigation, intravitreal antibiotic injection, and conjunctival resection at another hospital. Surgical management was chosen for this patient. During surgery, the filtering bleb was incised at the base of the fornix to expose the sclera, the preserved sclera was trimmed to adequately cover the choroidal herniation, and then sutured with 10-0 nylon suture. The avascular area of the conjunctiva was excised, rotated forward, and sutured with 10-0 nylon suture to complete the procedure (Figure 5).

**Figure 6:**
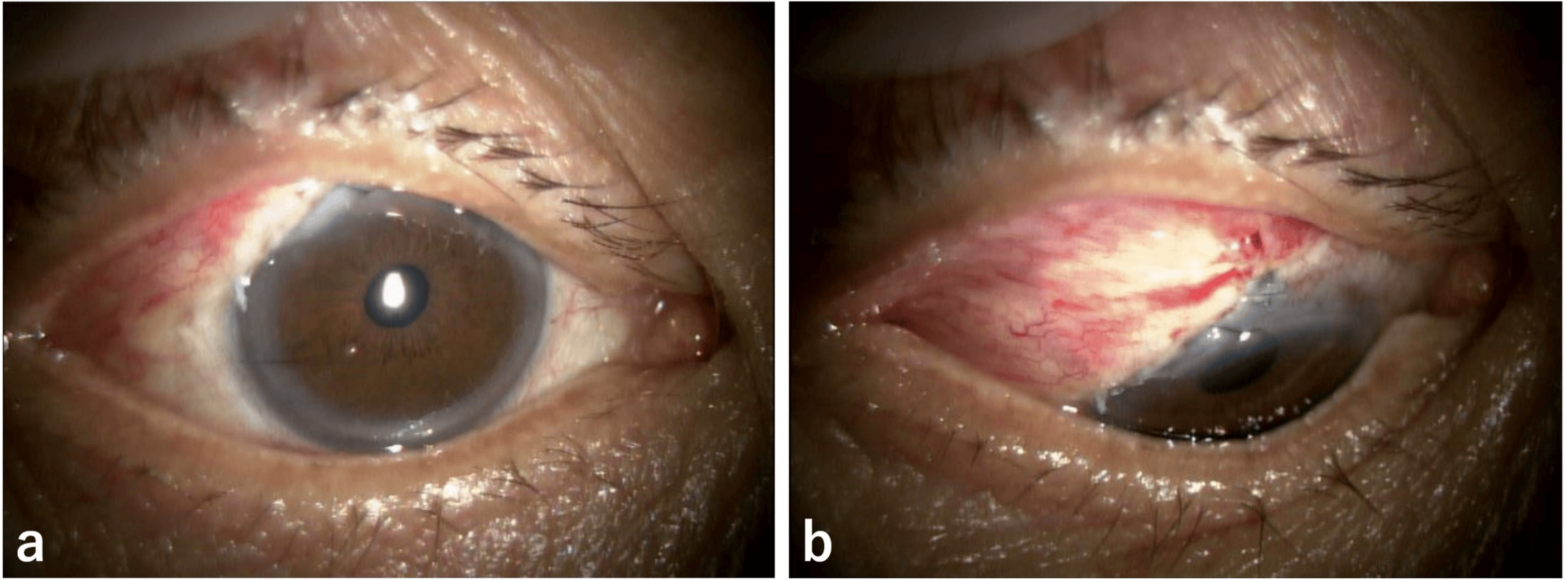
Postoperative slit-lamp photographs showing resolution of the conjunctival and scleral injury (a, b).

Postoperatively (Figure 6), aqueous humor leakage and choroidal detachment disappeared, and 0.1% betamethasone was administered four times daily and 0.3% gatifloxacin four times daily. The next day, the right IOP was 44 mmHg, so oral acetazolamide 250 mg twice daily and 0.005% latanoprost eye drops, along with 0.1% dorzolamide/0.05% timolol combination eye drops, were initiated. Two days after surgery, the pressure dropped to 27 mmHg and to 12 mmHg on the third day. However, it rose again to 20 mmHg on the sixth day and did not decrease thereafter, so reoperation was performed on the 24th day after surgery. 

**Figure 7:**
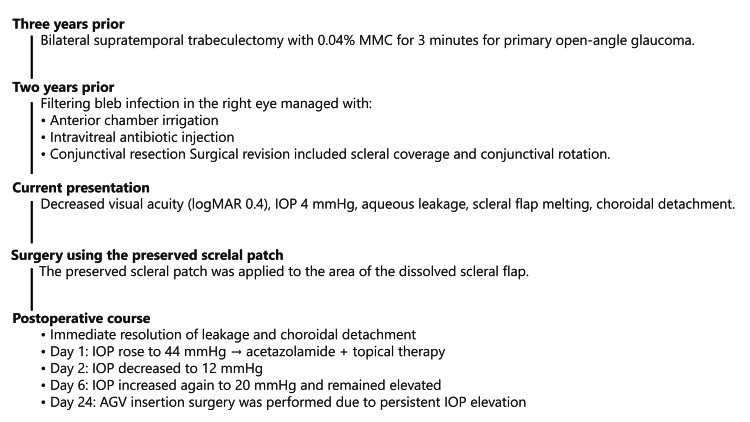
Clinical course of the Case 2. IOP, Intraocular Pressure; AGV, Ahmed Glaucoma Valve; MMC, Mitomycin C

Thus, on postoperative day 24, Ahmed Glaucoma valve™ (FP7, New World Medical, Inc., Rancho Cucamonga, CA, USA) insertion surgery was performed. The clinical course is summarized in Figure 7. The main body of the Ahmed Glaucoma valve was fixed to the inferotemporal side, and the tube tip was inserted into the ciliary sulcus. Postoperatively, the IOP remained stable at about 12 mmHg over one year with an eye drop.

## Discussion

The surgical treatments for scleral damage or fragility caused by infection, trauma, postoperative complications, or scleral softening [5,6] typically include preserved scleral patching [7,8], preserved corneal grafting [9], autologous scleral grafting [10], and amniotic membrane transplantation [11]. In cases of localized damage (e.g., trauma), amniotic membrane grafting is another viable treatment option [11]. However, repair of scleral damage following filtration surgery often requires coverage of a relatively wide area with full-thickness scleral tissue. Since the conjunctiva and amniotic membrane are generally insufficient for reinforcing strength, additional coverage with structurally robust tissues (e.g., cornea or sclera) is usually necessary.

Bakhsh et al. reported two cases of excessive filtration after trabeculectomy, wherein corneal grafts were implanted to achieve complete closure, resulting in successful outcomes [10]. Meanwhile, Shinozaki et al. reported two cases of surgical treatment for bleb infection after trabeculectomy combined with MMC [8]. These involved excising the infected bleb, removing necrotic scleral tissue, and suturing a preserved scleral graft. Additionally, a free conjunctival flap, harvested from the contralateral eye, was transplanted. Postoperatively, the IOP was well-controlled with topical and systemic antibiotics, plus prophylactic administration of glaucoma eye drops, with no recurrence in either case.

As presented in the current report, cases of scleral flap damage after trabeculectomy generally require repair using either scleral or corneal tissue. The available graft options include autologous scleral flaps, preserved cornea, and preserved sclera, as summarized in Table 1.

**Table 1:** Comparison of preserved scleral transplants, autologous sclera transplants, and corneal transplants.

Graft Type	Graft Source	Availability	Coverage for Large Defects	Immunogenicity	Ease of Reoperation
Preserved Scleral Transplantation	Preserved donor sclera	Requires donor tissue but can be preserved	Possible	Low	Easy
Autologous Scleral Transplantation	Patient’s own sclera	Limited (depends on anatomical conditions)	Limited	None	Limited
Corneal Grafting for Scleral Reinforcement	Donor cornea	Available via eye bank	Limited	Low	Limited (depends on graft availability)

Both sclera and cornea are primarily composed of extracellular matrix components, such as collagen, proteoglycans, and glycoproteins, which confer sufficient mechanical strength, making them suitable as materials for reinforcement [12]. As preserved tissues, they also carry a low risk of immune rejection. Autologous scleral grafts offer the advantage of minimal postoperative inflammation and virtually no risk of rejection. However, only a limited amount of tissue can be harvested due to thinning at the donor site, and full-thickness grafting is not feasible, resulting in limited structural reinforcement. Meanwhile, preserved corneal tissue provides adequate strength and is available in various sizes that can sufficiently cover the typical extent of scleral flap damage after trabeculectomy, making it a viable grafting material. However, corneal tissue is generally procured for corneal grafting for scleral reinforcement and is typically not intended for scleral repair. Lastly, preserved sclera can be obtained in multiple segments from a single donor globe after corneal harvesting; these segments can be trimmed into various sizes, allowing for flexible application in repairing scleral defects of different sizes.

In this study, we performed scleral flap melting repair and preserved scleral patching of the defect in eyes that underwent trabeculectomy with MMC. The damaged area improved in both cases, but high IOP occurred in the second case, which was managed with Ahmed Glaucoma valve insertion.

In the second case, elevated IOP was noted on the day after surgery. Although glaucoma eye drops were promptly administered, the IOP remained poorly controlled. Aside from the bleb closure, the secondary IOP elevation may have been triggered by the postoperative steroid eye drops or inflammation induced by FP-receptor agonists [13]. Therefore, in such cases, the risk of postoperative ocular hypertension must be carefully considered. When the IOP cannot be adequately controlled with topical medications, additional surgical intervention should be considered. Since repeat filtration surgery at the same site is generally impractical and carries a risk of hypotony, tube shunt surgery may be a more viable option.

MMC is frequently used in conjunction with trabeculectomy and is known to improve surgical outcomes [14]. However, it also carries a risk of scleral melting. As patient longevity increases, the incidence of late-onset scleral flap damage or melting can increase over the long-term postoperative course.

Preserved scleral grafts are considered effective for scleral repair due to their high biocompatibility, since they originate from the same tissue. However, their use remains limited, with only a few published reports. In such cases, it is likely that preserved corneal tissue provided by eye banks was used as a substitute. As previously mentioned, preserved sclera can be harvested in multiple segments from a single donor globe and stored at room temperature [15]. Nevertheless, preserved scleral patching may be a promising treatment option for scleral damage following trabeculectomy. Although not commonly used, preserved sclera warrants consideration as an alternative graft material to the cornea.

## Conclusions

Preserved scleral patches may represent a viable and effective treatment option for scleral damage resulting from trauma, infection, or MMC-associated scleral melting following trabeculectomy. In both cases presented, the use of preserved sclera led to successful anatomical repair and resolution of complications such as aqueous humor leakage and choroidal detachment. These outcomes suggest that preserved sclera offers sufficient mechanical strength and biocompatibility for reinforcing damaged scleral tissue. However, it is also necessary to consider the possibility that the effect of the filtering surgery may diminish over time, requiring additional glaucoma surgery. Furthermore, the limited number of cases and the absence of long-term outcome data warrant cautious interpretation of these findings.

Despite its limited use in clinical practice, preserved sclera has several advantages over other graft materials. It can be harvested in multiple segments from a single donor globe, stored at room temperature, and trimmed to match the size and shape of the defect. Compared to autologous scleral grafts, which are limited by donor site thinning and cannot provide full-thickness coverage, preserved sclera offers a more robust and scalable solution. While preserved corneal tissue is also strong and widely available, its use is typically reserved for corneal grafting rather than scleral reinforcement, making preserved sclera a more appropriate choice for scleral repair. Given these benefits, preserved scleral patching should be considered a valuable alternative graft material in the management of late-onset scleral flap injuries after glaucoma surgery, while acknowledging its limitations and the need for ongoing monitoring and larger studies to validate these results.
